# Chronic kidney disease duration and suicide risk among maintenance hemodialysis patients in China

**DOI:** 10.1093/ckj/sfae055

**Published:** 2024-03-13

**Authors:** Xinyue Wang, Xinmei Hao, Mi Ma, Wei Jiang, Baoshuang Li, Yan Xu, Ping Sun

**Affiliations:** School of Mental Health, Jining Medical University, Shandong, China; Department 2 of Elderly, Qingdao Mental Health Center, Shandong, China; Department 2 of Elderly, Qingdao Mental Health Center, Shandong, China; Department of Nephrology, the Affiliated Hospital of Qingdao University, Shandong, China; Department of Nephrology, the Affiliated Hospital of Qingdao University, Shandong, China; Department of Nephrology, the Affiliated Hospital of Qingdao University, Shandong, China; Department of Nephrology, the Affiliated Hospital of Qingdao University, Shandong, China; Department 2 of Elderly, Qingdao Mental Health Center, Shandong, China

**Keywords:** chronic kidney disease, CKD, suicide, suicide risk

## Abstract

**Background:**

Our aim was to investigate the relationship between chronic kidney disease (CKD) duration and suicide risk among maintenance hemodialysis patients in China.

**Methods:**

Patients with end-stage renal disease (ESRD) who received MHD were enrolled. The demographic and disease characteristics of MHD patients were collected using a self-designed basic information questionnaire. The Suicide Risk Assessment Scale was used to assess suicide risk.

**Results:**

A total of 543 (40.8%) patients had suicide risk with Nurses’ Global Assessment Scale for Suicide Risk scores ranging from 1 to 19 points. After adjusting for age, gender, disease conditions and mental state, the odds ratios of different CKD duration for suicide risk were 1.00, 2.02, 3.03 and 2.71, respectively (*P* for trend <.001). There were significant interactions between CKD duration and ESRD duration in relation to suicide risk (*P* for interaction <.001). There were also interactions between CKD duration and hemodialysis treatment duration, and suicide risk (*P* for interaction = .01). Patients with ESRD duration of ≤28 months or hemodialysis treatment duration of ≤24 months had the highest risk of suicide when the duration of CKD was 63–94 months, about 2–10 times higher than the other time groups.

**Conclusions:**

We found that CKD duration was associated with an increased risk of suicide in maintenance hemodialysis patients in China, independently of other risk factors. Early ESRD and maintenance hemodialysis were associated with suicide in CKD patients.

## Introduction

Chronic kidney disease (CKD) is a progressive disorder with irreversible dysfunction of the kidneys. The global incidence of CKD is increasing at a rate of 8% per year [1], and it is expected to become the fifth most common cause of early death in humans by 2040 [2]. Patients with end-stage renal disease (ESRD), also known as Stage 5 CKD, are characterized by uremia. Approximately 30% of CKD patients progress to ESRD, at which point the prolonged course of the disease is accompanied by a poor prognosis.

Renal replacement therapy is the main treatment for kidney disease. Due to the shortage of renal resources, high risk and cost of kidney transplantation, postoperative complications and post-transplantation rejection, maintenance hemodialysis (MHD) has become the mainstream choice for maintaining renal function in patients with ESRD. The mortality of MHD patients is 6.5–7.9 times higher than that of the general population [3]. MHD patients require long-term disease management and rely on machines for dialysis which, coupled with economic burdens, diet, activity restrictions and life pressure, seriously affects their quality of life.

MHD patients also suffered from mental and physical problems [4]. A large number of MHD patients also have mental disorders, especially depression and anxiety [5]. The prevalence of depression is about 46% in MHD patients, and suicide is the most serious result [6]. In 1971, a related study in the USA found that the suicide rate in dialysis patients was 400 times higher than that in the general population [7]. Persistent suicide risk increased the risk of death and other negative events in dialysis patients [8]. In addition, suicide causes huge economic losses, massive social burdens, and irreparable damage to individuals and families [9].

Currently, research on the psychological state of MHD patients in China mainly focuses on depression and anxiety, and there is little about the risk of suicide and related factors in ESRD patients on hemodialysis. Therefore, this study used a standardized scale to assess the risk of suicide in patients on MHD and develop targeted interventions in the early stages of the disease to improve the quality of life and reduce mortality in MHD patients.

## MATERIALS AND METHODS

### Subjects

We recruited ESRD patients treated with MHD in the Department of Nephrology, the Affiliated Hospital of Qingdao University Medical College from September 2019 to August 2021. The inclusion criteria included: (i) patients diagnosed with ESRD and CKD stage 5, whose glomerular filtration rate (GFR) was <15 mL/min/1.73 m^2^; (ii) disease stabilized, MHD ≥3 months duration; (iii) age ≥18 years old; and (iv) patients able to complete questionnaires and scales independently. Exclusion criteria were as follows: (i) acute renal damage caused by any disease; (ii) complicated with severe infection and other underlying diseases; (iii) stroke patients unable to express clearly; and (iv) history of mental illness or mental retardation. Finally, a total of 1330 MHD patients participated in this study.

### Clinical interview assessment

Three investigators, professionally trained in standardized assessment scales, assessed the severity of symptoms of subjects. Before obtaining informed consent from the subjects, the investigators explained in detail the purpose, methodology and significance of this study. The investigators did not make any tendentious or inductive prompts except for necessary explanations. After the subjects made their choices, the investigators confirmed and recorded the options. All questionnaires were filled out, proofread and collected one by one on-site to ensure the authenticity and quality of the questionnaires.

A self-designed basic information questionnaire was used to investigate the basic profile of MHD patients, including demographic characteristics (gender, age), disease status (duration of CKD, ESRD and MHD) and coexisting diseases. The Nurses’ Global Assessment Scale for Suicide Risk (NGASR) was used to assess the suicide risk of MHD patients. In this study, we defined those patients with an NGASR score of 0 as non-suicide risk patients. Depressive symptoms were assessed by the 17-item Hamilton Depression Rating Scale (HAMD-17), and patients with scores >7 were diagnosed with depression. The severity of anxiety symptoms was assessed using the 14-item Hamilton Anxiety Rating Scale (HAMA), and patients with scores of 0–7, 8–14, 15–21 and ≥22 were indicated as having non-anxiety, and mild, moderate and severe anxiety symptoms, respectively.

### Statistical analysis

The Kolmogorov–Smirnov one-sample test was used to detect the normal distribution of all variables. To compare differences between groups, the Mann–Whitney U test and analysis of variance (ANOVA) were used for non-normally distributed variables. In addition, chi-square tests were used for categorical variables. We used multivariate logistic regression to estimate the odds ratios (ORs) for suicide risk, adjusting for covariates including age, gender, disease status and mental status. Likelihood ratio tests (LRTs) were used to test for interactions between the duration of CKD and other factors and to compare the log-likelihood values of the two models with and without interaction terms. Restricted cubic spline regression was used to model the relationship between CKD duration as a continuous variable and suicide risk.

Data were analyzed and plotted using IBM SPSS 25.0 statistical software and R 4.1.2. *P*-values were set as two-tailed with a significance level of α = 0.05.

## RESULTS

A total of 1330 patients with MHD were included in our analysis. The mean age of the patients was 58.2 years (21–97 years) with 795 males and 535 females. A total of 543 (40.8%) patients had suicide risk with NGASR scores ranging from 1 to 19 points. Table 1 gives the characteristics of the patients by quartiles of CKD duration. MHD patients with a long duration of CKD had higher age, longer duration of ESRD and hemodialysis treatment, and higher HAMD scores, HAMA scores and NGASR scores.

**Table 1:** Characteristics of participants by CKD duration (in quintiles).

	CKD duration (months)				
Variables	Q1 (*n* = 336) (<30)	Q2 (*n* = 336) (30–62)	Q3 (*n* = 326) (63–94)	Q4 (*n* = 332) (>94)	*P*
Age	57.1 ± 14.1	57.8 ± 14.3	57.7 ± 12.5	60.1 ± 12.9	.02
Gender (%)					.11
Male (*n* = 795)	63.7	62.2	57.4	55.7	
Female (*n* = 535)	36.3	37.8	42.6	44.3	
End-stage renal disease duration (months)	16.4 ± 16.5	42.0 ± 17.7	56.5 ± 29.3	111.6 ± 61.5	<.001
Hemodialysis duration (months)	14.6 ± 10.3	39.1 ± 15.4	54.2 ± 31.0	105.8 ± 61.6	<.001
HAMD score	4.8 ± 5.0	4.8 ± 4.5	5.0 ± 4.7	6.2 ± 5.5	<.001
HAMA score	5.3 ± 6.8	5.7 ± 6.6	7.5 ± 7.7	6.2 ± 7.0	.001
NGASR score	0.8 ± 2.0	1.0 ± 1.7	1.3 ± 2.1	1.3 ± 2.4	.01

Data are presented as mean ± standard deviation or %.

We analyzed the relationship between duration of CKD and suicide risk (Table 2). In the model adjusting for age and gender, the ORs for suicide risk with increasing duration of CKD were 1.00, 1.62, 2.48 and 1.77, respectively (*P* for trend <.001). In the multivariate model adjusting for age, gender, disease conditions and mental state, the ORs for suicide risk were 1.00, 2.02, 3.03 and 2.71, respectively (*P* for trend <.001).

**Table 2:** Association between CKD duration and suicide risk.

			OR (95% CI)	
CKD duration (months)	Normal controls	Suicide risk cases	Age, gender adjusted	Multivariate^a^
Quintile 1 (<30)	237 (70.5%)	99 (29.5%)	1.0	1.0
Quintile 2 (30–62)	200 (59.5%)	136 (40.5%)	1.62 (1.18–2.24)	2.02 (1.40–2.91)
Quintile 3 (63–94)	160 (49.1%)	166 (50.9%)	2.48 (1.80–3.41)	3.03 (2.04–4.49)
Quintile 4 (>94)	190 (57.2%)	142 (42.8%)	1.77 (1.28–2.44)	2.71 (1.64–4.47)
*P* for trend			<.001	<.001

^a^Adjusted for age, gender, ESRD duration, hemodialysis duration, HAMD score and HAMA score.

**Figure 1:**
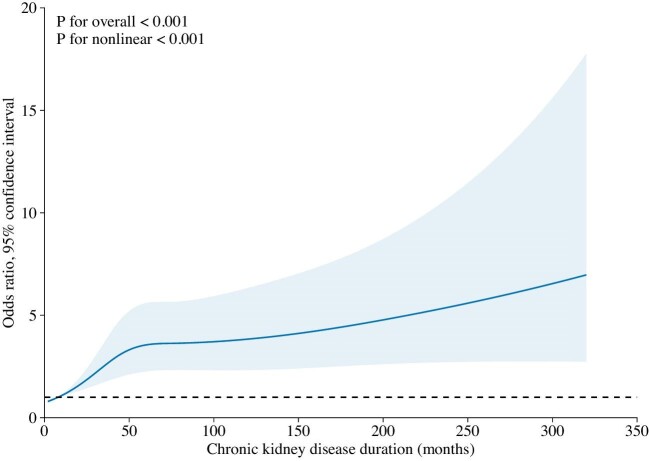
Restricted cubic spline of CKD duration and suicide risk.

We used restricted cubic splines to successively model the associations (Fig. 1). The regression splines suggested that CKD duration at about 7 months may have a threshold effect on the risk of suicide, with the effect leveling off at about 65 months and then increasing slowly. During this period, CKD duration was linearly related to suicide risk.

Then, we assessed the interaction between the CKD duration and other disease conditions (ESRD duration, hemodialysis treatment duration) and performed stratified analyses (Table 3). Considering the ability of the stratified analysis, we categorized the stratified factors, ESRD duration and hemodialysis treatment duration, into three categories (tertiles): low, medium and high levels.

**Table 3:** Stratified associations between CKD duration and suicide risk by age and clinic characteristic.

	CKD duration (months)					
	Q1	Q2	Q3	Q4	*P* for trend	*P* for interaction
Age						
<45 (*n* = 229)	1.0	2.22 (1.04–4.74)	2.48 (1.13–5.45)	2.56 (1.12–5.85)	.02	.24
45–60 (*n* = 492)	1.0	1.38 (0.82–2.34)	2.44 (1.46–4.05)	1.24 (0.72–2.12)	.12	
>60 (*n* = 609)	1.0	1.64 (1.02–2.65)	2.52 (1.55–4.08)	2.00 (1.26–3.17)	.001	
ESRD duration (months)						
Short, ≤28 (*n* = 459)	1.0	1.52 (0.80–2.88)	13.48 (6.61–27.50)	2.58 (1.10–6.05)	<.001	<.001
Median, 29–63 (*n* = 429)	1.0	5.38 (0.66–43.60)	6.76 (0.81–56.46)	6.57 (0.77–56.46)	.16	
Long, >63 (*n* = 442)	1.0	3.00 (0.15–59.89)	0.68 (0.09–4.91)	0.71 (0.10–5.11)	.75	
Hemodialysis duration (months)						
Short, ≤24 (*n* = 446)	1.0	1.87 (1.00–3.53)	11.02 (5.85–20.78)	1.83 (0.86–3.90)	<.001	.01
Median, 25–61 (*n* = 445)	1.0	1.23 (0.62–2.47)	1.56 (0.70–3.49)	1.47 (0.63–3.44)	.26	
Long, >61 (*n* = 439)	1.0	0.50 (0.03–8.95)	0.34 (0.03–3.80)	0.37 (0.03–4.14)	.95	
HAMD score						
Non, ≤7 (*n* = 991)	1.0	1.79 (1.21–2.65)	2.90 (1.97–4.27)	1.72 (1.14–2.59)	.001	.52
Depression, >7 (*n* = 339)	1.0	1.46 (0.76–2.82)	1.98 (1.01–3.91)	1.36 (0.74–2.47)	.31	
HAMA score						
Non, ≤7 (*n* = 925)	1.0	1.48 (0.99–2.21)	1.75 (1.16–2.65)	1.93 (1.30–2.87)	.001	.30
Low anxiety, 8–14 (*n* = 173)	1.0	2.00 (0.81–4.94)	2.15 (0.86–5.38)	0.79 (0.34–1.84)	.45	
Median and high, >14 (*n* = 232)	1.0	1.77 (0.79–3.96)	4.45 (2.01–9.84)	2.58 (1.09–6.11)	.003	

We found significant interactions between CKD duration and ESRD duration in relation to suicide risk (*P* for interaction <.001). The association between CKD duration and suicide risk was more pronounced in the short-duration group (*P* < .001) compared with the intermediate-duration group (*P* = .16) and the long-duration group (*P* = .75). There were also interactions between CKD duration and hemodialysis treatment duration, and suicide risk (*P* for interaction = .01), with a more pronounced correlation between CKD duration and suicide risk in the short-treatment duration group (*P* < .001) compared with the medium-treatment duration group (*P* = .26) and the long-treatment duration group (*P* = .95). We also assessed the interaction between CKD duration and mental state (depression, anxiety). The interaction test for HAMD scores and HAMA scores was non-significant (*P* for interaction was 0.52 and 0.30, respectively).

**Figure 2:**
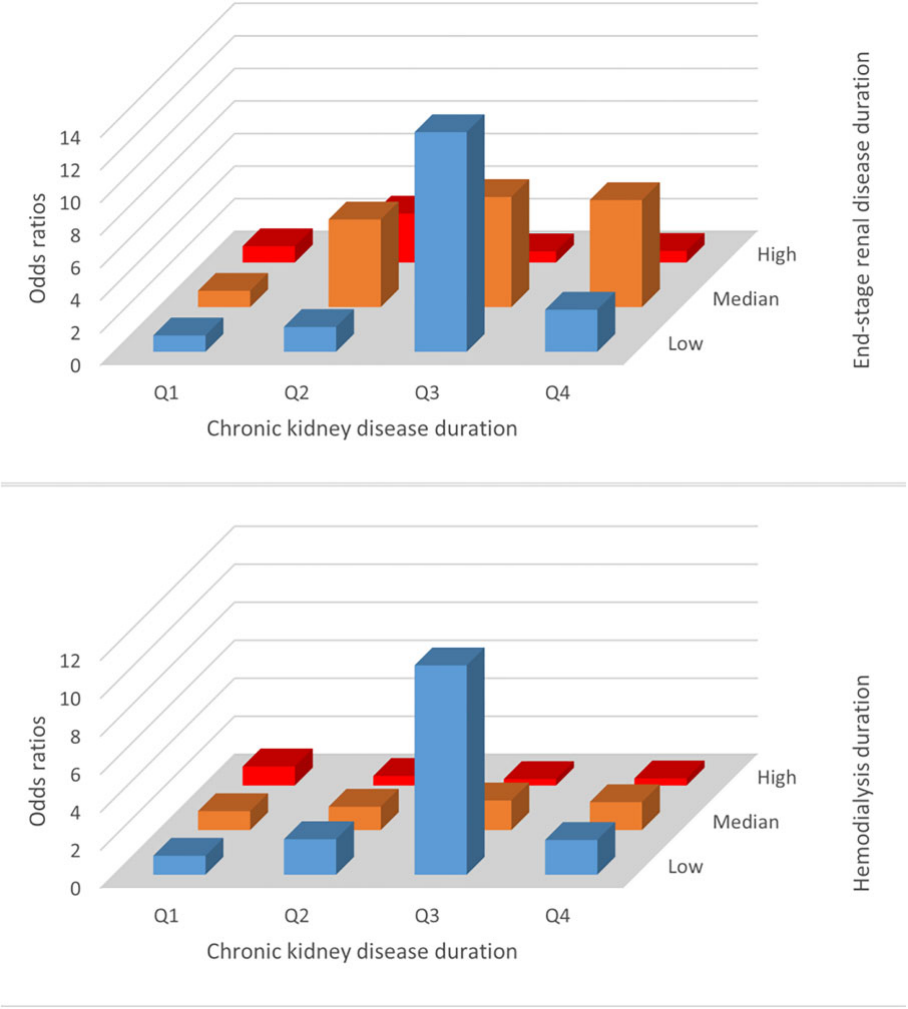
The suicide risk in different time groups.

In addition, we examined the combined effects of CKD duration and other disease conditions (ESRD duration, hemodialysis treatment duration) on suicide risk (Fig. 2). We found a pronounced phenomenon that when the CKD duration was 63–94 months, the suicide risk was the highest level for both short-term ESRD and short-term hemodialysis treatment.

## DISCUSSION

In recent years, medical models underwent a great change from the single biological model to the biological–social–psychological model, and the psychological and behavioral problems of non-psychiatric patients gained more and more attention. However, the psychological and behavioral problems of somatic patients are still neglected in general hospitals in China [10].

### The relationship between CKD duration and suicide risk

CKD is the beginning of ESRD, and hemodialysis is the primary treatment for ESRD. We found that the scores of anxiety, depression and suicide gradually increased with the prolongation of CKD. People with anxiety or depression are frequently suicidal, so the definition of CKD duration as an independent risk factor for suicide risk needs to be treated with caution [11]. Liu *et al*. found that suicide was associated with age, male, low education, alcohol or drug dependence, and recent hospitalization for psychiatric disorders in patients with MHD [12]. After controlling for these factors, they found an enhanced positive association between CKD and suicide risk. This is consistent with our results. We found a significant association between the duration of CKD and suicide risk in MHD patients. This association was independent of age, gender, duration of ESRD, duration of hemodialysis treatment and changes in scores of anxiety and depression.

Some studies have found that the number of suicide attempts in CKD patients is significantly higher than in the general people [13, 14]. A recent survey based on the Taiwan National Health Insurance Research Database (NHID) showed that after controlling for risk factors such as other chronic diseases, the suicide risk in CKD patients was 1.25 times higher than that in the control group [12]. Although previous studies have repeatedly demonstrated that suicide is a common problem in CKD patients, the risk of mental health problems in the early stages of CKD has rarely been investigated. Jhee *et al*. categorized CKD patients into six groups based on estimated glomerular filtration rate and found that the risk of suicide attempts was significantly increased in the relatively early (asymptomatic renal insufficiency) stages of CKD, and that the prevalence of suicide attempts increased as renal function further deterioration [15]. In this study, we found a linear relationship between the duration of CKD and elevated risk of suicide by counting the duration of illness in CKD patients, especially when the CKD duration was 7–65 months.

### Impact of other factors on CKD duration and suicide risk

In our study, patients with ESRD duration of ≤28 months or hemodialysis treatment duration of ≤24 months had the highest risk of suicide when the duration of CKD was 63–94 months, about 2–10 times higher than the other time groups. A study has found that ESRD patients are still significantly more likely to commit suicide than the general population after adjustment [13]. They also found that the suicide risk in ESRD patients was highest in the first 3 months of hemodialysis treatment and gradually decreased over time. Similarly, Chen *et al*. found that the suicide risk was highest in the first year of hemodialysis treatment and declined and finally stabilized, with the highest suicide risk in the first 6 months, especially the first 3 months [16]. ESRD patients are susceptible to depressive symptoms at dialysis initiation, which may explain the rapidly increasing risk of suicide early in hemodialysis treatment [17]. Some studies have agreed on the highest suicide risk being at <3 months of hemodialysis treatment, but Liu *et al*. found that patients on dialysis for >24 months also had a higher suicide risk than controls [12]. This is contrary to our study. Most studies have examined the risk factors, length of hemodialysis treatment and renal function status for suicide in ESRD patients, but few studies have reported on the effect of ESRD duration on suicide [12, 13, 15, 16].

The prevalence of depression in pre-dialysis CKD patients and dialysis patients has been reported to be much higher than in the general population, at about 2% to 4% [18, 19]. In addition, the degree of depression has been considered as a key factor influencing suicide behaviors [20, 21]. Our study found that depression was a risk factor for suicide, but did not have any interaction with CKD duration.

So far, there are few studies on suicide risk in patients with early CKD or ESRD, and further research is needed to determine whether appropriate management can reduce the suicide rate in CKD or ESRD patients. Our study suggests that except for the assessment and intervention of suicide in patients with early ESRD or MHD, we also need to pay attention to patients with CKD for 5–8 years, in which both early ESRD and MHD have a strong effect of increasing the risk of suicide. In addition, the focus on mental state is important for the management of MHD patients.

There are some limitations of this study. First, this study is a single-center cross-sectional study without long-term follow-up data, and it is limited by the sample size, study population and regional culture. It is not clear whether the results of this study are applicable to other countries. Second, this study only focused on the basic demographics, illness duration and coexisting diseases, and did not collect detailed information on education, income, family social support, laboratory-related data, and analysis of other relevant factors and confounders. Therefore, the modeling adjustments may not be sufficient in our study. Finally, this study only used the NGASR scale to assess suicide risk, ignoring subjective suicidal ideation and history of suicide in CKD patients.

## CONCLUSIONS

We found that CKD duration was associated with an increased risk of suicide in MHD patients in China, independently of other risk factors. The third quartile of CKD duration was associated with a considerable (2-fold) increase in suicide risk. Early ESRD and MHD were associated with suicide in CKD patients. The causality of these associations needs to be further confirmed in a multi-center longitudinal and prospective study.

## Data Availability

The datasets used and analysed during the current study are available from the corresponding author on reasonable request.
